# Translational Value of the Transdermal Administration of Bergamot Essential Oil and of Its Fractions

**DOI:** 10.3390/pharmaceutics14051006

**Published:** 2022-05-07

**Authors:** Damiana Scuteri, Laura Rombolà, Michele Crudo, Chizuko Watanabe, Hirokazu Mizoguchi, Shinobu Sakurada, Kengo Hamamura, Tsukasa Sakurada, Luigi Antonio Morrone, Paolo Tonin, Giacinto Bagetta, Maria Tiziana Corasaniti

**Affiliations:** 1 Pharmacotechnology Documentation and Transfer Unit, Preclinical and Translational Pharmacology, Department of Pharmacy, Health and Nutritional Sciences, University of Calabria, 87036 Rende, Italy; giacinto.bagetta@unical.it; 2 Regional Center for Serious Brain Injuries, S. Anna Institute, 88900 Crotone, Italy; patonin18@gmail.com; 3 Preclinical and Translational Pharmacology, Department of Pharmacy, Health and Nutritional Sciences, University of Calabria, 87036 Rende, Italy; laura.rombola@unical.it (L.R.); michelecrudo@virgilio.it (M.C.); luigi.morrone@unical.it (L.A.M.); 4 Department of Physiology and Anatomy, Faculty of Pharmaceutical Sciences, Tohoku Medical and Pharmaceutical University, 4-4-1 Komatsushima, Aoba-ku, Sendai 981-8558, Japan; w-chizu@tohoku-mpu.ac.jp (C.W.); mizo@tohoku-mpu.ac.jp (H.M.); 5 First Department of Pharmacology Fukuoka, Daiichi College of Pharmaceutical Sciences, Fukuoka 815-8511, Japan; s-sakura@tohoku-mpu.ac.jp (S.S.); k-hamamura@daiichi-cps.ac.jp (K.H.); tsukasa@daiichi-cps.ac.jp (T.S.); 6 Department of Health Sciences, University “Magna Graecia” of Catanzaro, 88100 Catanzaro, Italy; mtcorasa@unicz.it

**Keywords:** essential oil of bergamot, clinical translation, decolored phytocomplex, deterpenated phytocomplex, limonene, linalool, linalyl acetate, pain, formalin test, transdermal route

## Abstract

The essential oil of bergamot (BEO) has consistently proven antinociceptive and antiallodynic properties. Accordingly, the analgesic efficacy of the decolored essential oil (DEC), with higher levels of limonene, and the deterpenated (DET) fraction, with higher levels of linalool and linalyl acetate, was investigated using a formalin test after inhalation. The present study was aimed at characterizing the effects of BEO, its components with the highest pharmacological activity (represented by linalool, limonene, and linalyl acetate), and its DEC and DET fractions on the formalin test after transdermal administration relevant to clinical translation through topical application. To this aim, the schedule of intervention involved administration immediately after formalin injection or as a 5 min pretreatment followed by washout in ddY-strain mice. This study demonstrates, for the first time, the significant analgesic effect of all three constituents in the first and second phases, accounting for the efficacy of the essential oil in the formalin test. While all fractions revealed equal activity toward the phytocomplex in the early phase, the reduction in time of licking/biting during the late phase was more markedly induced by DEC. Moreover, pretreatment with BEO and its fractions followed by washout did not produce a significant reduction in licking/biting time in both phases of formalin-induced nociceptive response.

## 1. Introduction

The essential oil of bergamot (BEO) is obtained by cold pressing of the epicarp and, partly, of the mesocarp of the fresh fruit of bergamot (*Citrus bergamia* Risso et Poiteau). Of particular interest is its most abundant fraction (93–96%), i.e., the volatile one including oxygenated compounds, such as linalool and linalyl acetate, and monoterpenes and sesquiterpenes, such as limonene [1,2], known to be the most pharmacologically active components. On the other hand, the nonvolatile fraction includes bergapten, a significant photoactive compound displaying phototoxicity comparable to xanthotoxin (8-MOP) [3]. Recent analytic methodologies combining solid-phase microextraction with two-dimensional comprehensive gas chromatography–olfactometry–mass spectrometry identified the above three constituents to be key volatile aromatic active compounds [4]. In fact, the extraction of limonene, linalool, and linalyl acetate has been highly studied, and novel techniques, such as enzyme-mediated pervaporation, have been suggested [5]. At variance with most studies on essential oils in pain [6], the translational clinical efficacy and safety of BEO into the clinic is supported by its strong preclinical evidence of analgesic efficacy. This was achieved through studies meeting the best criteria for in vivo basic research, according to the guidelines for Animal Research: Reporting In Vivo Experiments (ARRIVE) [7], the Systematic Review Center for Laboratory Animal Experimentation (SYRCLE) risk of bias (RoB) tool [8], and the Collaborative Approach to Meta-Analysis and Review of Animal Data from Experimental Studies (CAMARADES) checklist for study quality [9]. In fact, this explains why, in spite of the potential for preclinical efficacy of various essential oils [10], the methodological flaws, the use of routes of administration [11], and the use of experimental pain models not relevant to the clinic (e.g., the acetic acid-induced writhing) have prevented their effective clinical translation. This is in agreement with the finding that the treatment of chronic pain mainly through aromatherapy has not yet been corroborated [12]. On the contrary, several mechanisms involved in the antinociceptive and antiallodynic properties of BEO are suggestive of suitability for clinical translation. For instance, BEO modulates glutamatergic transmission [13], involved in aging and neurodegeneration [14,15], and enhances morphine-induced analgesia [16]. These pharmacological actions make BEO an important candidate for the improvement of efficacy of opioids, which are the strongest analgesics but usually poorly effective in neuropathic pain [17] or pain of central nervous system origin [18]. Furthermore, metabotropic glutamate receptors mGluR7 and mGluR8 are implicated in pain descending pathway modulation [19]. BEO demonstrated an anti-allodynic effect in nerve ligation models of neuropathic pain [20], including after long-term application by means of an osmotic pump, resembling clinic chronic pain therapy [21]. To confirm the sound rationale for BEO clinical investigation, its analgesic efficacy was also revealed by the formalin test, particularly relevant to the clinic for its biphasic nature including both local nociceptive response and secondary central sensitization, typical features of chronic pain [22] which receives limited relief in real-world settings [23,24,25]. Indeed, 72% of elderly patients [26,27] and up to 80% of dementia patients in nursing homes [28] suffer from chronic pain. BEO inhalation exhibited preclinical analgesia in the formalin test [29], as well as clinic anxiolytic properties [30]. The analgesic efficacy for inhalatory administration of the whole phytocomplex, of its decolored (DEC) and deterpenated (DET) fractions, and of the main components endowed with pharmacologic activity, i.e. linalool, d-limonene, and lynalil acetate, on formalin-induced licking/biting was previously determined by our group [31]. BEO inhalation demonstrated a higher analgesic effect than its fractions [29], but the highest volume (800 µL) of the DEC fraction was as effective as comparable volumes of the whole phytocomplex [31]. On the contrary, the DET fraction proved inferior efficacy even at the highest volume [31]. Nanoemulsion of essential oils after topical transdermal administration resulted effective for peripheral and central analgesia in the hot plate test [32]. Therefore, the present original research was aimed at investigating, for the first time, whether the transdermal administration of BEO, its DEC fraction, with higher levels of limonene, its DET fraction with higher levels of linalool and linalyl acetate, and its components d-limonene, linalool, and lynalil acetate is endowed with an analgesic effect on formalin-induced licking/biting biphasic behavior. In fact, in agreement with the previous experiments, all fractions were defurocoumarinized to avoid phototoxicity.

## 2. Materials and Methods

### 2.1. Reagents

Formalin (36% solution; Kanto Chemical, Tokyo, Japan) dilution in saline solution was performed immediately before use up to a final concentration of 2%.

### 2.2. Phytocomplexes and Components

BEO and its fractions were obtained from Capua Company 1880 S.r.l. (Campo Calabro, Reggio Calabria, Italy). The chromatographic analysis conducted by the supplier according to the provided certificate of analysis reports the following composition of the batch of BEO used in this research: d-limonene (39.60%); linalyl acetate (31.09%); linalool (9.55%); bisabolene (0.45%); bergapten (2783.33 ppm); bergamottin (26,931 ppm). K peaks (%) of the defurocoumarinized essential oil were as follows: limonene 39.60; linalool 9.55; linalyl acetate 31.09; bisabolene 0.45. The decolored fraction (DEC) contained higher levels of d-limonene (44.00%) and lower levels of linalyl acetate, linalool, and bisabolene (28.23%, 4.45%, and 0.31%, respectively) than the entire phytocomplex. K peaks (%) of the DEC fraction were as follows: limonene 44.00; linalool 4.45; linalyl acetate 28.23; bisabolene 0.31. The deterpenated fraction (DET) differed from BEO by its lower levels of d-limonene (7.58%), higher levels of linalyl acetate (44.44%) and linalool (39.83%), and higher amount of bisabolene (0.61%). Limonene (CAS No 5989-27-5) was bought from Sigma-Aldrich (Sigma-Aldrich Chemical Co., St. Louis, MO, USA), while linalyl acetate (CAS No 115-95-7) and linalool (CAS No 78-70-6) were provided by TCI (Tokyo Chemical Industry Co. Ltd., Tokyo, Japan). K peaks (%) of the DET fraction were as follows: limonene 7.58; linalool 39.83; linalyl acetate 44.44; bisabolene 0.61. The phytocomplexes and their components were diluted in Jojoba oil (CAS No 61789-91-1, Wako Pure Chemical Industries Ltd., Osaka, Japan).

### 2.3. Animals

The animals used for this research study were male mice ddY (Japan SLC, Hamamatsu, Japan) of 23–26 g of weight. They were housed in individual cages under a 12 h light/12 h dark cycle at room temperature of 22–24 °C with 55% ± 5% relative humidity. The mice were provided with food and water ad libitum. The experiments followed the Ethical Standards for Investigation of Experimental Pain in Animals Guidelines, and they were approved by the Animal Care and Use of Tohoku Medical and Pharmaceutical University Committee from 8 January 2014 for minimizing the suffering of animals and for using the minimum number of animals necessary for reliable results. In agreement with G*power sample size calculation [33], according to previous studies [29,31], the minimum number of animals to achieve reliable statistically significant results was *n* = 8 per experiment.

### 2.4. Pain Model

The experimental pain model selected was the formalin test, since it consists of an early phase due to the nociceptive stimulation followed by a late phase of pain behavior induced by central sensitization and affected by aging [22]. The test required 1 h of habituation in a transparent cage sized 22.0 × 15.0 × 12.5 cm. After this period of acclimatation, a volume of 20 μL of formalin (2% in saline) was administered through intraplantar (i.pl.) injection to the mice, using a microsyringe with a 26-gauge needle. The time of licking/biting, which was the pain behavioral indicator considered, was monitored with a handheld stopwatch in 5 min intervals. The early phase started immediately after the i.pl. injection of formalin and lasted for 10 min (0–10 min), while the late phase began at the end of the first 10 min and lasted up to 30 min.

### 2.5. Experimental Design

**Figure 1:**
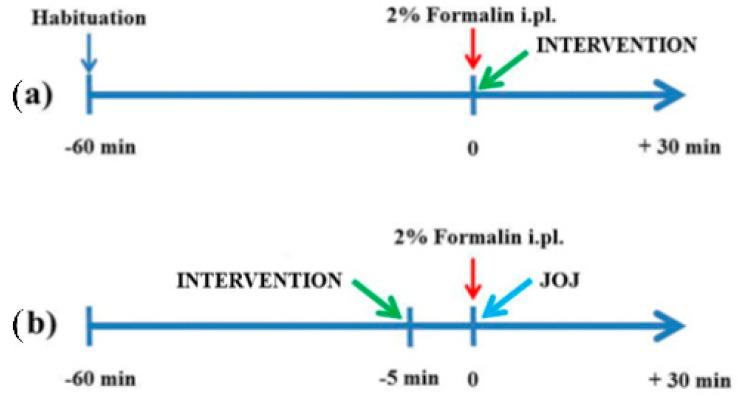
Experimental protocol. (**a**) posttreatment; (**b**) pretreatment. Schedule of transdermal administration of the intervention (essential oil of bergamot and its fractions and main pharmacologically active components) or of the vehicle (Jojoba oil, JOJ) during the time course of the formalin test.

The effects of transdermal administration of the entire phytocomplex BEO and of its components linalool, limonene, and linalyl acetate, as well as of the DEC and DET fractions, on licking/biting due to formalin injection were investigated through a double-intervention protocol. All the fractions were defurocoumarinized to avoid phototoxicity. As the purpose of the present research was to assess the anti-nocifensive efficacy of BEO and of its DEC and DET fractions, as well as of its main volatile components when administered transdermally in a pain model relevant to clinic, two points of administration were established: (a) in the first experimental group, the intervention (BEO/DEC/DET) was applied on the plantar surface immediately after (A) formalin injection (BEO-A/DEC-A/DET-A); (b) in the second experimental setting, the duration of the effect of the intervention after washout was examined by applying the intervention (BEO/DEC/DET) on the plantar surface 5 min before (PRE) formalin administration (BEO-PRE/DEC-PRE/DET-PRE), removing it through absorbent paper just before the i.pl. injection of the algogen compound, followed by the transdermal application of Jojoba oil (JOJ), i.e., the vehicle. The third experimental group received the transdermal administration of a solution of 44% d-limonene (equal to the percentage present in the DEC fraction)/39.8% linalool (equal to the percentage contained in the DET fraction)/44.5% linalyl acetate (equal to the percentage contained in the DET fraction) in JOJ immediately after i.pl. formalin injection. The volume of all the interventions and of the vehicle administered was 10 µL. The experimental schedule is illustrated in Figure 1.

### 2.6. Statistical Analysis

Data were reported as the mean ± SEM of the time of licking/biting and evaluated statistically for differences by one- or two-way ANOVA, followed by Bonferroni’s test, considering *p* < 0.05 as statistically significant.

## 3. Results

### 3.1. Analgesic Efficacy of the Transdermal Topical Treatment with BEO on Formalin-Induced Licking/Biting Biphasic Nocifensive Behavior

**Figure 2:**
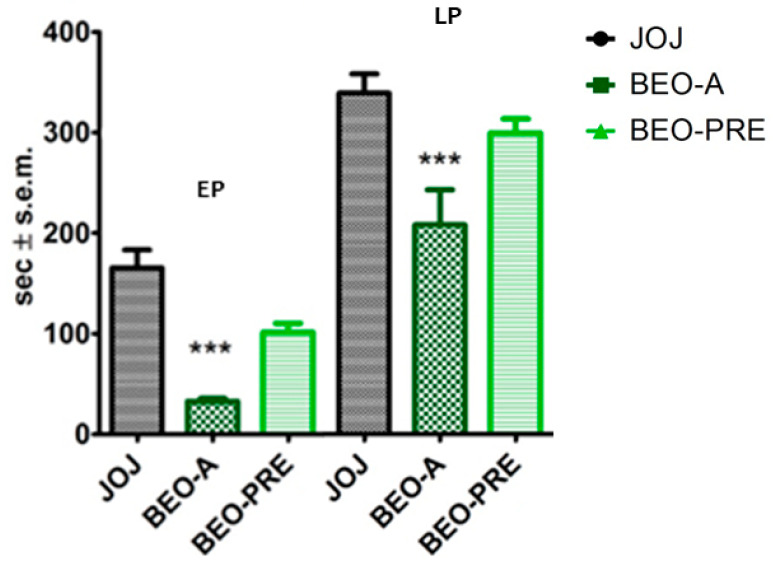
Analgesic effect of the transdermal administration of the essential oil of bergamot (BEO) on licking/biting behavior in the formalin test. The essential oil of bergamot (BEO) reduced licking/biting behavior when administered immediately after formalin injection in the early and in the late phase of formalin test (BEO-A). This effect did not persist after the 5 min pretreatment followed by washout (BEO-PRE). Overall licking/biting time is expressed as time in s ± SEM during the early phase (EP) (0–10 min) and the late phase (LP) (10–30 min). One-way ANOVA followed by Bonferroni test; *n* = 8; *** *p* < 0.001.

The transdermal administration of 10 µL of the whole phytocomplex BEO immediately after the i.pl. injection of 2% formalin solution (BEO-A) induced a significant reduction in the licking/biting time in both the first and the second phase of the nociceptive response during the formalin test (*p* < 0.001). On the other hand, the pretreatment, consisting of the topical application of 10 µL of pure BEO on the paw site of the injection 5 min prior to the i.pl administration of 2% formalin, followed by the removal of BEO and subsequent application of JOJ (BEO-PRE), did not produce a significant reduction in the licking/biting time in the first and second phase of the nociceptive response induced by formalin. These data are reported in Figure 2.

### 3.2. Analgesic Effect of BEO, DEC and DET Fractions on Formalin Test after Transdermal Administration

**Figure 3:**
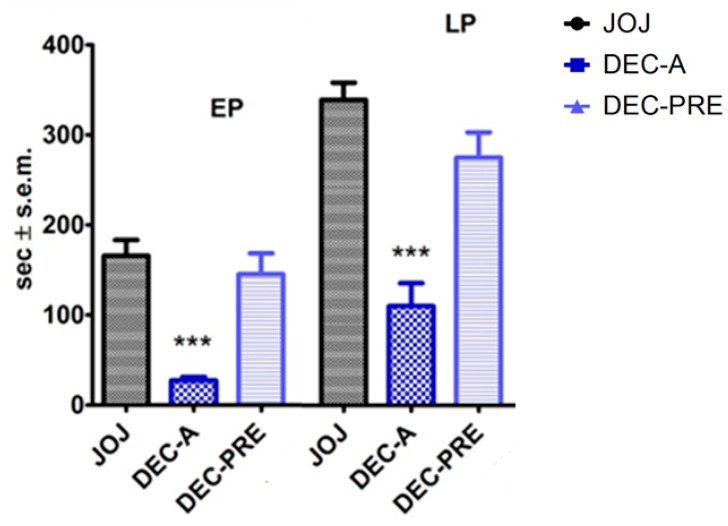
Analgesic effect of the transdermal administration of the decolored fraction of the essential oil of bergamot (DEC), enriched in d-limonene, on licking/biting behavior in the formalin test. The decolored fraction of the essential oil of bergamot (DEC), enriched in d-limonene, produced a reduction in the licking/biting behavior when administered immediately after formalin injection in the early and in the late phase (EP, LP) of the formalin test (DEC-A). The latter analgesic effect did not last after the 5 min pretreatment followed by washout (DEC-PRE). Overall licking/biting time is expressed as time in s ± SEM during the EP (0–10 min) and the LP (10–30 min). One-way ANOVA followed by Bonferroni test; *n* = 8; *** *p* < 0.001.

Similarly to what was observed for the entire phytocomplex, the topical application of 10 µL of DEC fraction solution immediately following the i.pl. injection of formalin (DEC-A) produced a significant reduction in the licking/biting time in both the early and the late phase (EP, LP) of the formalin test (*p* < 0.001). In this case, the topical pretreatment 5 min before formalin administration and followed by washout and vehicle application did not evoke a significant decrease in the time of licking/biting in any of the two phases of the formalin test. The results of DEC transdermal administration are displayed in Figure 3.

**Figure 4:**
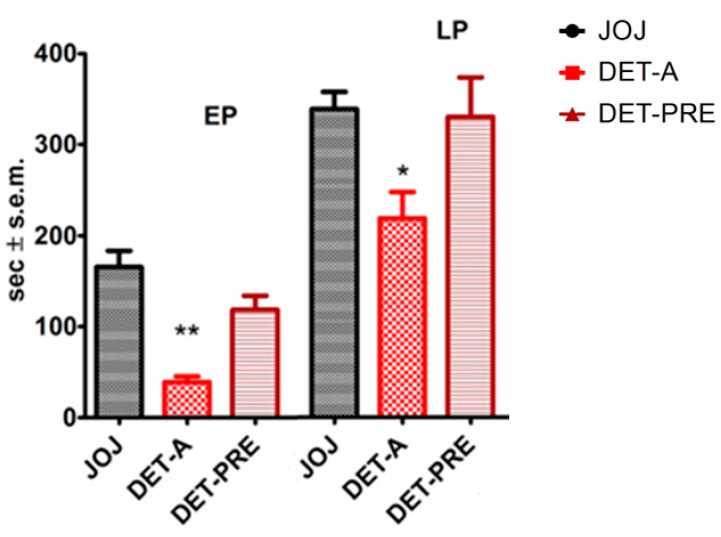
Analgesic effect of the transdermal administration of the deterpenated fraction of the essential oil of bergamot (DET), enriched in linalyl acetate, on licking/biting behavior in the formalin test. The deterpenated fraction of the essential oil of bergamot (DET), enriched in linalyl acetate, transdermally administered immediately after formalin injection (DET-A), decreased the licking/biting time in the early and in the late phase (EP, LP) of the formalin test. Administered as 5 min pretreatment followed by washout (DET-PRE) and application of JOJ, the DET fraction did not reduce the licking/biting time in s. Overall licking/biting time is expressed as time in s ± SEM during the EP (0–10 min) and the LP (10–30 min). One-way ANOVA followed by Bonferroni test; *n* = 8; * *p* < 0.05, ** *p* < 0.01.

The DET fraction of BEO, enriched in linalyl acetate, administered after formalin injection (DET-A) significantly reduced the nocifensive response of licking/biting in the first (*p* < 0.01) and in the second phase (*p* < 0.05) of the formalin test. It is interesting to notice that this proved to be the least pharmacologically active fraction in terms of reversal of licking/biting behavior in formalin test. Moreover, as described for the whole phytocomplex and for the DEC fraction, the effect of 5 min pretreatment with DET waned after its removal and replacement with JOJ vehicle. The results are reported in Figure 4.

### 3.3. Effects of the Transdermal Administration of d-limonene, Linalool, and Linalyl Acetate on Formalin-Induced Licking/Biting Behavior

**Figure 5:**
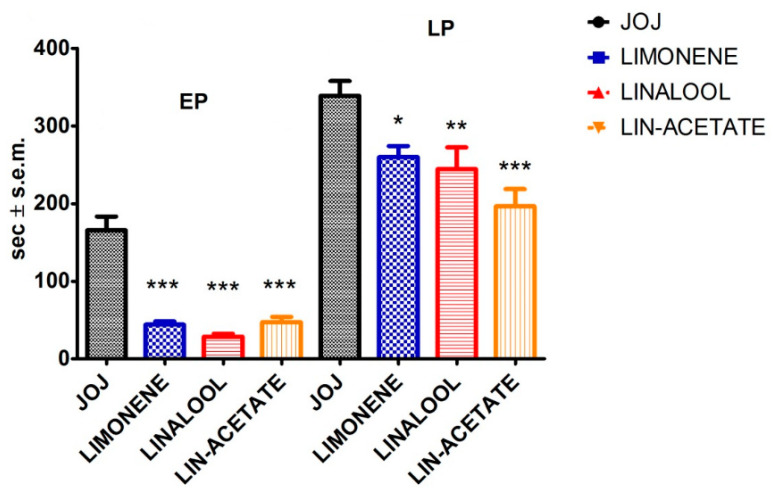
Analgesic effect of d-limonene, linalool and linalyl acetate on licking/biting behavior in the formalin test. The main pharmacologically active components of the volatile fraction of the essential oil of bergamot (d-limonene, linalool, and linalyl acetate), transdermally administered immediately after formalin injection, significantly decreased the licking/biting time in the early and in the late phase (EP, LP) of the formalin test. All these constituents were almost equally effective in the EP, while linalyl acetate was the most active followed by linalool and limonene in the LP. Overall licking/biting time is expressed as time in s ± SEM during the EP (0–10 min) and the LP (10–30 min). One-way ANOVA followed by Bonferroni test; *n* = 8; * *p* < 0.05, ** *p* < 0.01, *** *p* < 0.001.

To unveil the contribution of the main individual constituents of the volatile fraction of BEO to the phytocomplex analgesic pharmacological activity when transdermally administered, the effects of d-limonene, linalool, and linalyl acetate were analyzed on the first and on the second phase of the formalin test and throughout the time course. The effects of d-limonene were tested by applying 10 µL of a solution of d-limonene at 44% (equal to the percentage of d-limonene in the DEC fraction) diluted in JOJ on the paw site of the injection, immediately after i.pl. injection of formalin. d-Limonene induced a significant reduction in the licking/biting time in the first (*p* < 0.001) and the second (*p* < 0.05) phase of the nociceptive response induced by the i.pl. formalin (Figure 5). To investigate the analgesic efficacy of the topical application, a volume of 10 µL of a 39.8% linalool solution (corresponding to the percentage of linalool present in the DET fraction), immediately after formalin, produced a significant decrease in the licking/biting time in the first (*p* < 0.001) and the second phase (*p* < 0.01) (Figure 5). Similarly, the transdermal administration of 10 µL of a solution of linalyl acetate at 44.5% (equal to the percentage of linalyl acetate present in the DET fraction) in JOJ immediately following the i.pl. injection of formalin produced a statistically significant reduction (*p* < 0.001) in the licking/biting time in both the early and the late phase of the nocifensive behavior induced by the i.pl. injection of formalin (Figure 5). According to the gathered results, linalyl acetate was the most active, followed by linalool and limonene in the late phase, while all these constituents were almost equally effective in the early phase.

**Figure 6:**
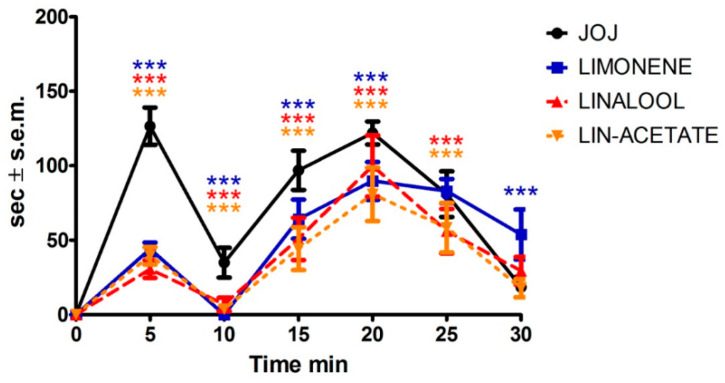
Time course of the analgesic efficacy of d-limonene, linalool, and linalyl acetate on licking/biting behavior in the formalin test. Effect of limonene, linalool, and linalyl acetate on the time course of the licking/biting time monitored at 5 min intervals of and expressed as time in s ± S.E.M reported during the formalin test. Two-way ANOVA followed by Bonferroni test; *n* = 8; *** *p* < 0.001.

The time course showed a marked and significant reduction (*p* < 0.001) in the licking/biting behavior observed between 0 and 20 min post injection by limonene (Figure 6). Moreover, linalool particularly exerted its analgesic efficacy at all time intervals between 0 and 25 min post injection (Figure 6). Linalyl acetate produced a noteworthy significant decrease in the licking/biting time up to 25 min after formalin administration (*p* < 0.001) (Figure 6).

## 4. Discussion

The present study demonstrates, for the first time, the analgesic efficacy of the transdermal administration of DEC and DET phytocomplexes and of the main components of BEO endowed with pharmacologic activity, i.e., d-limonene, linalool, and linalyl acetate, on the biphasic behavior of licking/biting induced by 2% formalin. While all the fractions were equally active to the whole phytocomplex in the early phase, the DEC fraction induced a more marked decrease in the nocifensive behavior represented by the time of licking/biting during the late phase. This is in agreement with findings previously observed for inhalatory administration, under which the highest volume (800 µL) of the DEC fraction was as effective as comparable volumes of the whole BEO [31], while even the highest volume of the DET fraction resulted less effective [31]. Moreover, all the constituents, i.e., d-limonene, linalool, and linalyl acetate, were almost equally effective in the early phase, while linalyl acetate was the most active followed by linalool and limonene in the late phase. This is inversely correlated with the effects of the latter constituents on spontaneous motor activity [31], linked to the capability of BEO to increase the immobility time in the open field and the forced swimming behavioral tasks [34], representing the attempt to escape anxiety [35]. Interestingly, all three main components exerted a significant analgesic effect in both the first and the second phase, accounting for the efficacy of the whole essential oil in this experimental pain model, selected precisely because it is characterized by a late phase of pain behavior induced by central sensitization and affected by aging [22]. In fact, the demonstration of the analgesic properties of the different fractions and of BEO components after transdermal administration is necessary for clinical use since BEO in the pharmaceutical form of a base cream incorporating a nanotechnological delivery system based on solid lipid nanoparticles [36] is under investigation in an active clinical trial for proof of concept of the management of severe dementia with agitation through analgesia (NCT04321889) [37]; this is fundamental since clinical studies for the treatment of several forms of pain often exclude cognitively impaired patients [38,39], not testing the most novel therapies [40] and not informing about the best analgesic practice in this fragile population subjected to physiological modifications and variability in drug response [41]. The DEC fraction was enriched with 44% limonene, confirming the previous observation supporting the role of d-limonene in analgesia. In particular, d-limonene was proven to reduce vimentin levels similarly to the autophagy inhibitor chloroquine [42], and this is important since alterations of the autophagic machinery have been widely reported to occur during pain conditions relevant to the clinic [43] and during dementia, supporting a role for drug repositioning [44]. In fact, agitation is often triggered by underdiagnosed and unrelieved pain [45], being treated with antipsychotics that increase death risk in the aged patients due to accidents of a cardiac and cerebrovascular nature [46], and this issue has worsened during the pandemic [47,48]. Another important feature of the pharmacological activity of BEO for clinical use is its lack of sedative effects (that could increase cognitive deterioration), supported by the involvement of serotonergic neurotransmission [49]. This neuromodulation is a fundamental target during aging and neurodegeneration [50], as demonstrated by the action of rapid-acting antidepressants [51], which could represent a revolutionary therapy for the neuropsychiatric symptoms [52]. Therefore, the deep characterization of the analgesic properties of BEO, as well as of its fractions and components, via the transdermal route is fundamental since its engineered nanotechnological delivery system, NanoBEO, is administered via the same route and is the soundest candidate essential oil for investigation in the clinical management of pain and agitation. From a pharmacokinetic perspective, the effect of 5 min pretreatment of the whole phytocomplex, as well as of the DEC and DET fraction, waned after removal and replacement with JOJ vehicle. In fact, the latter did not persist after the 5 min pretreatment followed by washout, supporting the need for the topical exposure to last over 5 min. Future studies will investigate the pharmacokinetics and herb–drug interactions [53] of NanoBEO, fundamental in case of polypharmacy [54], noteworthy for the elderly [55]. In fact, according to our findings, the pretreatment consisting of the transdermal administration of BEO or of its enriched fractions on the paw site of the injection 5 min before formalin injection, followed by washout, did not produce a statistically significant decrease in the formalin-induced licking/biting time both in the early and in the late phase. Therefore, the investigation of the time of exposure to the phytocomplex topically applied required for a long-lasting effect before washout represents a pharmacokinetic/pharmacodynamic relationship to be elucidated in future research.

## Data Availability

Data are contained within the article.
